# Asian American, Native Hawaiian, and Pacific Islander Health

**DOI:** 10.1089/heq.2022.29015.rtd

**Published:** 2022-12-26

**Authors:** Krystal Ka'ai, Monica McLemore, Zhuo (Adam) Chen, Grace X. Ma, Raynald Samoa, Thu Quach, Xinzhi Zhang

**Affiliations:** ^1^ Executive Director of the White House Initiative on Asian Americans, Native Hawaiians, and Pacific Islanders (WHIAANHPI) and the President's Advisory Commission on Asian Americans, Native Hawaiians, and Pacific Islanders (PACAANHPI), Washington, District of Columbia, USA.; ^2^ Editor in Chief, Health Equity, and Interim Director of the University of Washington's Center for Antiracism in Nursing, Seattle, Washington, USA.; ^3^ Associate Professor of Health Policy and Management, College of Public Health, The University of Georgia, Athens, Georgia, USA.; ^4^ Associate Dean for Health Disparities, Founding Director of Center for Asian Health, Laura H. Carnell Professor at Lewis Katz School of Medicine, and Director of Community-Based Research at Fox Chase Cancer Center at Temple University, Philadelphia, Pennsylvania, USA.; ^5^ Endocrinologist at the City of Hope, Duarte, California, USA. Member, President's Advisory Commission on Asian American, Native Hawaiians, and Pacific Islanders; Co-Chair of the Data Disaggregation Subcommittee.; ^6^ President of Asian Health Services, Oakland, California, USA.; ^7^ Chief of Health Inequities and Global Health Branch at the Center for Translation Research and Implementation Science at the National Heart, Lung, and Blood Institute (NHLBI), part of the National Institutes of Health (NIH), Bethesda, Maryland, USA.

## Expert Panel


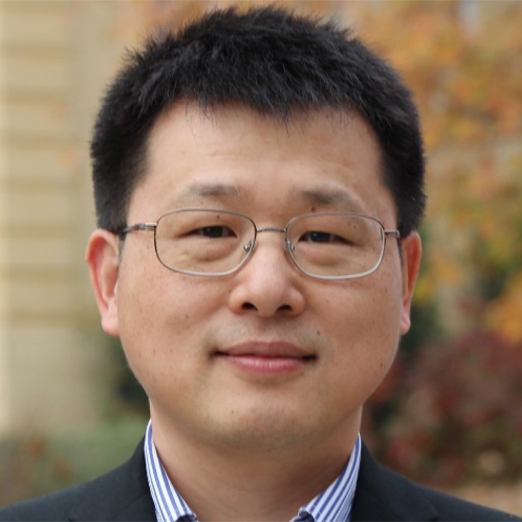


**Dr. Zhuo (Adam) Chen (moderator)** is associate professor and DrPH Program Coordinator, Department of Health Policy and Management, College of Public Health, University of Georgia (UGA), Athens, Georgia, USA; visiting professor of health economics and director of academics (0.2FTE), Centre for Health Economics, School of Economics, University of Nottingham Ningbo China. Dr. Chen leads the Interdisciplinary Approaches to Social Determinants of Health Pre-Seed Team at UGA and co-leads the Health Disparities Research Workgroup within the UGA College of Public Health. He earned his PhD in economics and MS in statistics from Iowa State University. Before Dr. Chen joined the UGA, he was a senior health economist with the U.S. Centers for Disease Control and Prevention (CDC). He has been a guest researcher since 2017 with the CDC Office of Genomics and Precision Public Health. Dr. Chen is affiliated with the Atlanta-based China Research Center and the Core China Research Group—Universidad de Navarra. Dr. Chen's current research interests include health economics, economics of genomics, social determinants of health, global health, health systems, mental health, and economic evaluation. He has published more than 100 peer-reviewed publications. His works have appeared in *Lancet*, *JAMA Network Open*, *Health Policy*, *Health Economics*, *Journal of Health Economics*, *Genetics in Medicine*, and *Social Science & Medicine*. He was a recipient of the CDC Excellence in Social and Behavioral Science Research Award in 2013 for his work on examining the role of geographic scale in testing the income inequality hypothesis. He earned the Excellence in Diversity Award (Civilian) from the Federal Asian and Pacific Americans Council in 2016 for his work promoting diversity and inclusion at CDC. He serves as an associate editor of *Health Equity*, academic editor of *PLoS One*, and on the editorial board of *Journal of Family and Economic Issues*, *China CDC Weekly*, and *Global Health Research and Policy*.


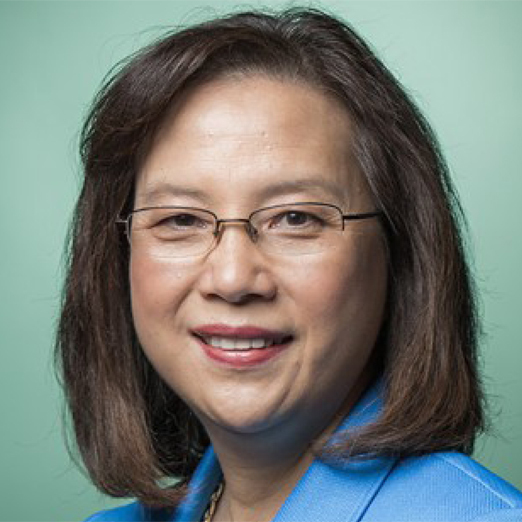


**Dr. Grace X. Ma** is associate dean for Health Disparities, founding director of Center for Asian Health, Laura H. Carnell Professor at Lewis Katz School of Medicine, and director of Community-Based Research at FCCC, Temple University. Dr. Ma is a nationally recognized public health scientist, leader, and pioneer in cancer and health disparities research among underserved vulnerable racial/ethnic minority populations. In 2000, she established Center for Asian Health among nation's first centers dedicated to reducing cancer and health disparities in underserved AAPI. Dr. Ma has made seminal contributions in improving health equity and reducing health disparities. Over the past 22 years, Dr. Ma as principal investigator has directed four cycles of NCI/NIH funded cancer health disparities research centers/networks in the U.S. east region of DC/MD, PA, NJ, and NYC, which established robust partnerships with over 400 community/faith-based organizations (CBOs) and clinical partners that predominately serve racial/ethnic minorities. Her community-based participatory research and patient-centered outcome research focus on improving early detection, patient navigation, cancer prevention and control (hepatitis-related liver cancer, cervical, breast, lung, and colorectal cancers) and access/quality of health care in underserved and racial/ethnic minorities. Dr. Ma has directed more than 100 intervention or observational longitudinal studies, including large-scale cluster randomized intervention trials, implementation and dissemination studies at worksites, community health centers, primary care clinics, CBOs, and churches. Dr. Ma has mentored over 260 junior faculties, postdoctoral, doctoral, and master minority fellows that created a pipeline of diverse workforce of researchers in health disparities. Dr. Ma currently cochairs NIH CEAL-AANHPI Interest Group.


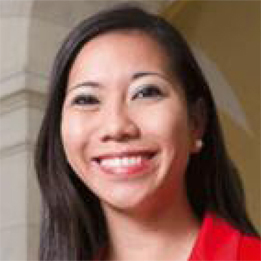


**Ms. Krystal Ka'ai** is the executive director of the White House Initiative on Asian Americans, Native Hawaiians, and Pacific Islanders (WHIAANHPI) and the President's Advisory Commission on Asian Americans, Native Hawaiians, and Pacific Islanders. In this role, she is responsible for advising the Biden administration on the coordination and implementation of federal programs and initiatives to advance equity, justice, and opportunity for Asian American, Native Hawaiian, and Pacific Islander communities. Prior to joining WHIAANHPI, Krystal worked on Capitol Hill for over a decade, including serving as the executive director of the Congressional Asian Pacific American Caucus (CAPAC) for 8 years. She previously held positions with the U.S. Senate Committee on Indian Affairs, the State of Hawai'i, Office of Hawaiian Affairs, and the National Japanese American Memorial Foundation. Krystal was born and raised in Hawai'i and is the first Native Hawaiian to ever lead WHIAANHPI.


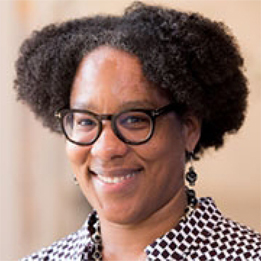


**Dr. Monica McLemore** is a tenured professor in the Child, Family, and Population Health Department and interim director for the Center for Anti-Racism in Nursing at the University of Washington School of Nursing. She retired from clinical work in 2019; however, currently provides flu and COVID-19 vaccines. Her research is focused on reproductive justice. Her peer-reviewed articles, op-eds, and commentaries have been cited in five amicus briefs to the Supreme Court of the United States and three NASEM reports. She became editor-in-chief of *Health Equity* in 2022.


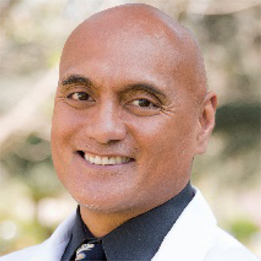


**Dr. Raynald Samoa** is a graduate from the University of Washington School of Medicine. He completed his residency and fellowship training at USC Los Angeles County General Hospital. He is currently an endocrinologist at the City of Hope. Dr. Samoa served as the lead for the National Pacific Islander COVID-19 Response Team and has authored several manuscripts describing the impact of COVID-19 on Pacific Islander communities. He has testified to the House of Representative Ways and Means Committee during a session entitled *THE DISPROPORTIONATE IMPACT OF COVID-19 ON COMMUNITIES OF COLOR.* Dr. Samoa serves on the President's Advisory Commission on Asian American, Native Hawaiians, and Pacific Islanders, and is the cochair of the Data Disaggregation Subcommittee. He currently is the technical advisory lead for the Healing Association of Pacific Islander Physicians and the Data and Research Council of the National Association of Pacific Islander Organizations.


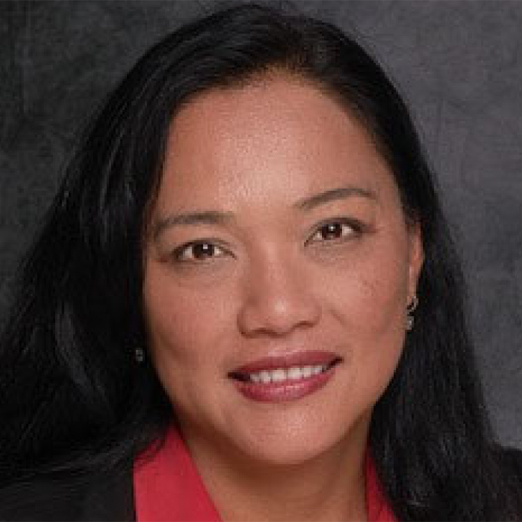


**Dr. Thu Quach** has worked in public health and health care for 25+ years. Her research, service, and advocacy work have been grounded in her own lived experience as a Vietnam refugee and the struggles her family faced in the health care system. Dr. Quach is the President of Asian Health Services (AHS), a federally qualified health center in Oakland serving 50,000 patients in English and 14 languages. She is involved in research and policy efforts to promote health equity, emphasizing the importance of language justice and data disaggregation. She has expanded culturally competent mental health services at AHS. In recent years, Dr. Quach has been leading the organization in addressing racial disparities in COVID-19, including starting up culturally and linguistically competent community testing sites, contact tracing targeting Asian Americans and Pacific Islanders (AAPIs), and vaccination efforts. In addition, she has been conducting groundbreaking research on COVID-19 impacts on the AAPI population and launched efforts to collect stories of language and digital barriers faced by the AAPI community during the pandemic. Her recent work has focused on responding to anti-Asian violence and addressing mental health needs for survivors. Dr. Quach received her bachelor of arts at UC Berkeley, master's in public health at UCLA, and PhD in epidemiology at UC Berkeley.


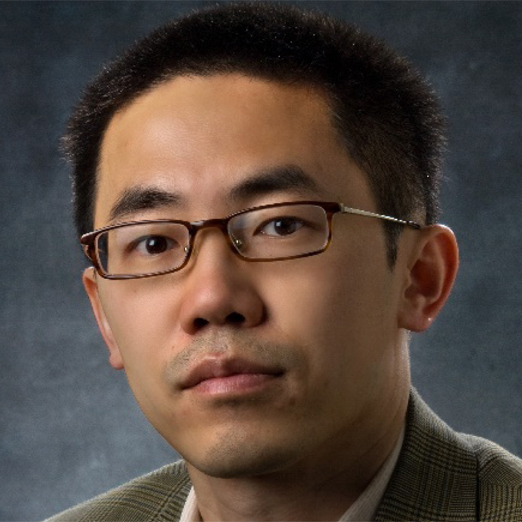


**Dr. Xinzhi Zhang** is the chief of Health Inequities and Global Health Branch at the National Heart, Lung, and Blood Institute (NHLBI). Prior to that, he was a program director in the National Center for Advancing Translational Sciences, National Institute on Minority Health and Health Disparities, and a senior medical epidemiologist at the Centers for Disease Control and Prevention. Dr. Zhang has authored 4 articles for inclusion in CDC's *Morbidity and Mortality Weekly Report*, 3 book chapters, and 73 articles published in peer-reviewed journals, including the *Journal of the American Medical Association.* He is one of the editors of the 2021 textbook on *The Science of Health Disparities Research* and served as the interim editor in chief of *Health Equity.* Throughout his career, Dr. Zhang has received many honors and awards from NIH, CDC, AHRQ, American Public Health Association, and U.S. Public Health Service, including Secretary's Award for Distinguished Service, Hubert H. Humphrey Award for Service to America, four NIH Director's Awards, two Presidential Unit Citations, and two Outstanding Service Medals. Dr. Zhang received his MD from Peking Union Medical College in 1998 and his PhD in health services administration from the University of Alabama at Birmingham in 2003.

***Dr. Chen:* My name is Adam Chen. I am an associate professor of Health Policy and Management at the College of Public Health at the University of Georgia. I would like to welcome you to this special session and thank you for joining the fireside chat on the special issue of *Health Equity*—Asian American, Native Hawaiian, and Pacific Islander (AA and NHPI) health. This special issue is a collection of short reports, research articles, perspectives, and editorials on AANHPI health.**

**Dr. Grace Ma and I are the guest editors for this special issue, along with Dr. Monica McLemore, the editor in chief of *Health Equity*. We really appreciate the support from the Blue Shield of California Foundation, the China Medical Board, and the American Foundation in promoting and disseminating research on AA and NHPI health.**

**Dr. Monica McLemore is a renowned scholar in reproductive justice. She is interim director of the Center for Anti-Racism in Nursing, Department of Child Family and Population Health at the University of Washington, and also the editor in chief of *Health Equity*. Before her move to the University of Washington, Dr. McLemore was the Thelma Shobe Endowed Chair and tenured associate professor with the University of California, San Francisco.**

**She retired from active clinical practice after a 28-year clinical nursing career in 2019. Her research addresses reproductive health and justice and has been cited in five amicus briefs to the Supreme Court of the United States and three National Academies of Science, Engineering, and Medicine reports.**

**Dr. McLemore:** Thank you, Dr. Chen. It is my distinct pleasure to be here and to be able to engage with this incredible group of scholars, clinicians, policymakers, and funders because this is a real treat.

I cannot take credit at all for this incredible issue because it was in process when I became the editor in chief of *Health Equity*. So, I do want to shout out the Mary Ann Liebert publishing staff who supported the journal, Dr. Grace Ma, who did a heavy lift with Dr. Adam Chen and his team to really be able to thoughtfully and mindfully go through the multitude of submissions that we receive for this special edition of our journal.

There are 15 different articles, not including our introductory editorial, which includes short reports. There are research articles. There are three different perspectives. There are several editorials on AANHPI health and health disparities. And it is very very important that we were very intentional around Asian American, Native Hawaiian, Pacific Islander—really disaggregating different groups.

We received articles from across the spectrum of individuals. And we got so many that we had to publish some in the original edition of *Health Equity* outside of the special edition because the quality and the caliber of these topics were so important to us that we wanted to make sure that they were amplified because the science that was behind these articles were really important. Many of the articles addressed health disparities.

They highlighted community-engaged solutions, and some highlighted key policy priorities that were necessary. And some really amplified and identified the shameful anti-Asian hate, violence, and other factors that this community has disproportionately borne. So, it is my great privilege and honor to know that we have in our midst Krystal Ka'ai, who is the executive director of the White House Initiative on AA and NHPIs, and is part of the Presidential Advisory Commission on Asian Americans, Native Hawaiians, and Pacific Islanders.

In this role, she is responsible for advising the Biden-Harris administration on the coordination and implementation of federal programs and initiatives to advance health equity justice and opportunities for AA and NHPI communities. Prior to joining the initiative, Krystal worked on Capitol Hill for over a decade, including serving as the executive director of the Congressional Asian Pacific American Caucus for 8 years.

She also previously held positions with the United States Senate Committee on Indian Affairs, the state of Hawaii and their office of Hawaiian Affairs, and the National Japanese American Memorial Foundation. Krystal was born and raised in Hawaii and is the first Native Hawaiian to ever lead the White House Initiative on AANHPI health.

I am so grateful that you chose to spend your time with us today, and I am very much looking forward with your discussion with Dr. Chen.

**Ms. Ka'ai:** Thank you so much, Dr. McLemore, for having me, and thank you to Dr. Chen. Aloha. It is so great to be here with all of you on behalf of the White House Initiative, as well as the President's Advisory Commission on Asian Americans, Native Hawaiians, and Pacific Islanders.

***Dr. Chen:* We are curious about the key priorities of the White House Initiative on Asian American, Native Hawaiian, and Pacific Island Health. Do you have any particular areas that the initiative is trying to push to work on?**

**Ms. Ka'ai:** Yes, absolutely. And I wanted to again thank you for this opportunity to present on behalf of the administration. Our White House Initiative on Asian Americans, Native Hawaiians, and Pacific Islanders has actually existed for over two decades. It was first created under the Clinton administration in 1999, and has been reauthorized under every administration, both Democratic and Republican administrations, ever since then.

It was most recently reauthorized and expanded to be its largest in scope in history under the Biden-Harris administration last May during AA and NHPI Heritage Month, during which the president signed Executive Order 14031, which is tasked with advancing equity, justice, and opportunity for our AA and NHPI communities. And for the first time in history, the name Native Hawaiian is explicitly listed in the Executive Order.

So, for me, as someone of Native Hawaiian descent, this is especially meaningful to be able to lead this initiative under its expanded and broadened charge. We are focused on a number of different priorities under this administration—everything from advancing health equity, of course, to addressing the alarming surge in anti-Asian violence and hate we have seen over the course of the pandemic, to looking at long-standing inequities, like the need for greater data disaggregation and language access for our extremely diverse communities that represent the broader AA and NHPI umbrella.

We also are focused on things like economic equity, educational equity, and housing—so many different components that really do tie very well into addressing social determinants of health. Environmental justice is also included in that. So, for us, the most immediate priorities have been to address the long-standing inequities and disparities that have really been exacerbated over the past 2.5 years of the COVID-19 pandemic.

We know that the AA and NHPI community, long before COVID, faced a number of health disparities. Like cancer rates, for instance, looking at diseases like viral hepatitis, and hepatitis B in particular, where our communities have been disproportionately impacted. But we also know that throughout COVID, unfortunately our communities were oftentimes overlooked because of the lack of disaggregated data, which oftentimes I think fed into this false narrative that Asian Americans, Native Hawaiians, and Pacific Islanders were not facing the same COVID-19-related health inequities as other communities of color, when in fact that is not true.

This is especially true for our Native Hawaiian and Pacific Islander populations. I see that we have one of our [President's Advisory Commission on Asian Americans, Native Hawaiians, and Pacific Islanders] Commissioners, Dr. Raynald Samoa, who is here and who will probably speak a little bit more to those findings. But, I think that was really a challenge for us from the federal government's perspective was being able to ensure that we were doing our best to have a really robust pandemic response, while also addressing the unique needs of diverse populations.

This includes making sure that we are doing all that we can to address the really unique needs of diverse communities that fall within the broader AA and NHPI umbrella, and in particular, our Native Hawaiian and Pacific Islander communities who faced some of the most significant hospitalization and mortality rates from COVID-19 out of any racial group. Not just among our broader AA and NHPI group, but even compared with other racial and ethnic groups.

I think a lot of the work we have been doing through our White House Initiative, as well as the President's Advisory Commission, has really been to highlight the unique disparities and needs of our diverse populations, and to ensure that we are doing all that we can as we continue to recover from COVID, to get appropriate resources.

To address some of those long-standing systemic challenges that have long plagued the community, we have focused on the need for greater data disaggregation to truly understand where those disparities are. Another priority is language access, given that about a third of our population is limited in English proficiency. There are so many different challenges that we are facing, I think, as a result of COVID that have really been exacerbated over the past 2.5 years.

Mental health is another one that we are really looking to address, especially in response to the rise in anti-Asian hate we have seen. We know that there are so many communities and individuals who are traumatized from everything that has happened over the past 2.5 years, and still do not feel safe in this country. And so there are a lot of different priorities that we are looking to tackle under this administration, and I'm very heartened by the commitment we have received from the very top, from our President and Vice President, who truly see our communities, who understand our communities, and have made sure that through our Initiative, as well as the Commission, that we have the resources that we need to tackle these challenges.

***Dr. Chen:* Thank you, Ms. Ka'ai. This is great. We really appreciate your efforts in promoting health of AA and NHPIs. My next question is how we can reduce the gaps and how we can improve the health among the most disadvantaged populations over this community?**

**Ms. Ka'ai:** That is an excellent question, Dr. Chen. And I think we are very fortunate. I was remiss. I did not mention this earlier, but our White House Initiative, despite its name, we are actually physically housed within the U.S. Department of Health and Human Services. And we are cochaired by two members of the President's cabinet, including HHS Secretary Xavier Becerra, as well as U.S. Trade Representative Ambassador Katherine Tai.

I think one unique advantage we have about being housed within HHS is really being able to look at how we are promoting health equity beyond just the course of this first term of the administration, and really taking that broader perspective of how we can create fundamental change that will have a lasting impact for decades and generations to come. We are working very closely with the Healthy People 2030 initiative, for instance.

We are also looking at ways to collaborate across the department—working with the Substance Abuse and Mental Health Services Administration on mental health issues; working with NIH on research; working with different components, like the CDC and so many others to really make sure that we are doing all that we can to uplift the challenges that our communities are facing, and also to ensure that we are getting adequate support to the communities, whether it is in the form of capacity building and grants that can go out to community health centers that are serving AA and NHPI populations, or other entities that are providing critical culturally and linguistically appropriate services and support to our populations.

We are also looking at things across the government. So beyond just HHS, we know that there are so many other components of the federal government that have equities in terms of doing all that we can to promote the health and safety of our communities. And so that is something we are doing—working with the Department of Justice, for instance, on ways to ensure safety and justice for our communities who have been impacted by hate crimes and violence; working with the Department of Education to promote not just educational attainment, but also looking at bullying in schools.

So, these are just a couple of the examples of how we are using our platform and our interagency coordination, as well as our external engagement with the general public, to really ensure that we are not only sharing resources from the federal government and getting that information out to the public, but also creating meaningful policy, programmatic, as well as outreach initiatives that can really help to get resources and support into the hands of our AA and NHPI communities all across the country.

**Dr. Ma:** Thank you so much for sharing all the initiatives that are happening at the Biden administration with your leadership and the team. I think this is really very important to address priorities and issues in our community. I think one of the things coming out of this pandemic is probably the silver lining of giving a little bit emerging visibility of our populations, AANHPI. Still, there is a long way to go, and we are really looking forward and looking up to the leadership and the resources to get our communities, and the science or lay communities capacity building so we can really help the community to reduce disparities.

**Dr. McLemore:** Ms. Ka'ai, I am so grateful for your office, for your work, for having the opportunity to engage with you, and to hear that the Biden-Harris administration has this in sharp focus on the radar.

In light of that, what are you excited about? What is on the horizon that you want this audience to know that the administration is working on?

**Ms. Ka'ai:** Well, there are so many things that it is hard to pick just one. But I do, again, really want to thank you, Dr. McLemore, for this opportunity, and also Dr. Chen. One of the things that I am most excited about that is actually really historic and unprecedented that is being done under this administration is this commitment that the President and Vice president made on Day 1 to advance racial equity throughout the entirety of our federal government.

President Biden signed an executive order on his first day in office to do this. And one of the key components of that racial equity Executive Order was the creation of an Equitable Data Working Group. I know this is not the most interesting of topics, per se, but for me, the data disaggregation work that is being done within the administration is truly historic. A lot of it is being done quietly, but there has also been a lot of robust engagement with community stakeholders, data scientists, and a number of folks internally within government, as well.

I am really excited about that, because we all know that we cannot really promote equity if we do not understand where the challenges are, where the disparities are. So that data are so critical, in my opinion, to our community—knowing that for so long, we have been historically lumped together and seen as a monolith, when in actuality, our AA and NHPI populations are so diverse and so robust.

Really being able to understand and drill down into the data and ensure that we have the appropriate tailored solutions to address so many of the significant disparities that have long plagued our various ethnic populations, I think, is really going to have lasting impacts for generations to come.

**Dr. McLemore:** I am so glad you brought that up. The data piece is hugely important. Throughout the 15 articles that we had in the special edition, this topic kept coming up—this issue of lumping communities together in large groups is not helpful in tailoring interventions, in being able to amplify community wisdom in order to have translation of our work.

We cannot intervene, we cannot fund, and we cannot really address things if we are not counting them, right?

**Dr. Quach:** I think that some of the problems that we always face—from data disaggregation to language access—have been decades long, but then there is a new problem as we face COVID, which is the digital divide. Is there work that is being done to address the digital divide that is compounding with language access and the visibility of our populations?

**Dr. McLemore:** Also, before you answer that, can you add on anything you can say about workforce development? That was going to be my question.

**Ms. Ka'ai:** Those are both very excellent questions, and I will do my best to tackle both. There has been a lot of work being done under this administration. And I think COVID, to your point, has really, again, highlighted so many of these long-standing inequities, one being also the digital divide. And we have seen—whether it was access to telehealth, or even just children being able to log on to do their schoolwork—how unfortunately communities of color, and especially those in our lower-income communities, were some of our hardest hit from the pandemic and were really disproportionately impacted.

This is something that the administration is working to tackle. We have literally infused trillions of dollars into the economy through a number of historic pieces of legislation—everything from the Inflation Reduction Act that the President just signed into law earlier this year, to the Bipartisan Infrastructure Deal, to the American Rescue Plan Act, and so much more. And so there have been a number of investments that have been made to really tackle so many of these inequities that were exacerbated throughout COVID, including increasing broadband access.

A ton of money was also infused into community health centers, as well as into telemedicine. Workforce diversity is also a key component. We know that if we are looking to the future, especially as we recover from COVID and look at ways to improve our systems overall, that we really do need to have a robust health care workforce, and one that is also culturally and linguistically competent.

Through a number of the pieces of legislation I mentioned earlier that have since been enacted, we have really made a concerted effort to ensure that we are targeting federal dollars, programs, and initiatives toward these efforts.

**Dr. McLemore:** Thank you so much. It has been a real gift having you here with us, and we are very grateful that you made the time to be able to participate in this fireside chat. I think the implications of this special edition are far and wide. And we are intensely grateful to know that the federal government is really using these data to inform policy that hopefully will result not only in improved health outcomes, but also in better quality of life. We are grateful that we had the first half hour with Ms. Ka'ai. We want to pivot because we have some of the actual authors here, which I am super grateful to now have an opportunity to moderate a discussion with the authors to really hear about why they wrote their pieces and how to use these findings. For those of you who are joining us who did not participate in our Twitter Spaces earlier this summer, we had an opportunity to hear from authors about their work, and why they wrote these articles, and how they want these data to be used.

The first question I have is, what motivated you to write your piece for this special edition? What was that passion that really drove you to want to write and submit this piece?

The second question is, what do you think are the most important findings? How do you want people to use your work? Particularly policymakers, funders, patients, people with lived experience. Adam, could you start us off with what motivated you to be the guest editor and what were your most important research findings?

***Dr. Chen:* Sure. Thank you, Dr. McLemore. Well, in terms of the opportunity to become a guest editor, I would probably point to Dr. XinZhi Zhang. We had a chat about the coming May, which is the Asian American, Native Hawaiian, and the Pacific Islander Heritage Month. And we were thinking of how to celebrate that and to address the issues. For example, the anti-Asian hate and how Asian American communities are suffering from the pandemic.**

**So, that is the reason that we started planning for the special issue. And I knew that Dr. Zhang and Dr. Grace Ma probably will have more on this, as well. For the other article that we had, using the health, ethnicity, and pandemic survey, which we call HEAP. During the pandemic, we have seen cases of anti-Asian hate.**

**We conducted a survey—over 2700 Americans and oversampled Asian Americans and African Americans. We tried to assess—what is the situation for the communities.**

**We did find 19% of Asian American reported being discriminated and had experienced hate crimes. And the percentage was 19% for both Asian Americans and African Americans, 15% for Hispanics, and about 3% for non-Hispanic whites. So, there is a big difference, and we need to work on that. I think community engagement, working with the community, working within the research community, and working with the policy makers, will be able to help to address these problems.**

**Dr. Ma:** Thank you so much for this opportunity. And to answer your question about motivation, in my whole entire career, I have been committed and devoted to reducing health disparities, primarily not only in the Asian American populations but also in other underserved groups. I see there are a lot of the disparities that have not been acknowledged, but it is in the community. The model minority has become an issue that is masking the disparities. Also, I had the opportunity to cochair a community-engaged alliance against COVID disparities of an AANHPI interest group. The article that is in this special issue is a collective voice. It is very important for basically representing the voices of the leaders across the country and really concerned about critical issues, what kind of framework that we often use, and to come back to studies, and conducting our community-engaged work.

The framework we come up is a cultural safety framework that we wanted to continue to embrace. We have seen these anti-Asian issues coming up during the pandemic. But how can we really tackle this racism at all levels? Sometimes, it may not be violent. It could be implicit bias in our health care system policies that are really reflecting the limited access to care and the resources that could be improving the well-being of the population.

Ms. Ka'ai just mentioned the language access. So, language access could be reflected in many areas, not only in our daily life. It could be in the justice system, could be in the health care access, treatment, prevention, et cetera. We wanted to really focus on the capacity building in terms of culturally, and linguistically appropriate research and community engagement. I think that is very important for us to continue to improve advanced health equity. And the other issue that we also addressed in this article is data disaggregation. What we are specifically concerned by is we do not have better tools to really disaggregate. We are really happy to see the leadership promoting data disaggregation.

But at the end of the day, when the researchers or our community come up with these research studies, where are the resources that can really cover in the different languages, and culturally compatible measurements that could capture meaningful data? Because there is so much diversity among the Asian Americans, Native Hawaiians, and Pacific Islanders. So, having better tools to have granular disaggregated data is so important. The last topic that we discussed in the article is leadership representation. I think all these issues can be informative for policy makers, but also needs a lot more resources to make it happen.

**Dr. Samoa:** So, what motivated us to write the article? We had every intent of publishing it. But I think probably what was more important was what was the intent behind gathering data? I think one of the long-standing issues that we have had with the employment of Pacific Island communities regarding health is that we have been constantly told that our communities were too small to look at, even though isolated reports showed that we had significant health disparities and chronic diseases, such as diabetes, cancer, and heart disease.

So, we had all this looming around us with absolutely no idea how to address them specifically because of the lack of disaggregated data. So, when COVID came around, we wanted to make sure that this was not going to be the excuse again, that statistical limitations were not going to be justification for the omission of health outcomes. Because what we found out in the pandemic—which is probably true before the pandemic—was *invisibility means death*.

And what was very interesting was when we started working with the national group, the American Association of Asian psychologists, we had to figure out how to overcome the obstacles that we have been told prohibited the reporting of NHPI data. So, we used a very culturally centered community-approved approach. We went to the community from the get-go. They were involved with the initial design of the study. Traditional sampling frame design does not work with Native Hawaiians and Pacific Islanders. We had to use one that was more community informed. We had to specifically focus on our community's expertise on regionality, on NHPI subgroups, on behavior. That was how we were able to come up with a study design that sampled what we wanted to sample.

The initial assessment was just to look at what was the experience of NHPI during the pandemic. From that, we were able to discern some things that were very important for us to fight COVID for these communities. Once we started to look at the vaccine hesitancy data, it became very clear as to what was prioritized that we needed to push out there to inform local health, state, and federal agencies as how to help support these communities.

One of the things we noticed was there were very common among other communities of color. The socioeconomic gradient that persists with a lot of disease outcomes—the more money you make, the more educated you are, the more protected you are. We saw a lot of that with some of our larger Pacific Islander communities. But it was not consistent.

There were times when the socioeconomic gradient did not hold up. It was not about filtering information through an economic and educational lens. It was filtering information, period. There were sections of the community that were—regardless of their income and their education—not partaking in protective health behaviors, or at least not likely to, based on their hesitancy.

When we were able to push that out, we were able to show that the NHPI community is not a monolith. You cannot have general outreach efforts based on the top three populations because you will miss a lot of the ones that are suffering as much. There are the Marshallese in Arkansas, there are the Tongans in Utah. And if you use a one-size-fits-all approach to all Native Hawaiians and Pacific Islanders, you are not going to move the needle very much. So, that was such a successful effort.

This community-based leadership within study design, dissemination, outreach is how I want that data to be used. When you come up with an excuse of, we have statistical limitations regarding anonymity, that we cannot report data—we can tell you that that is not true. Or that you can overcome those obstacles. And the community should be your first stop to overcome those obstacles. A lot of what we are looking at in regard to outreach in NHPI communities where they come from research, where they come from agency outreach, is aimed at academic centers, and using them to reach out to community organizations. They do not need to do that.

You can center your research and center your advocacy or your outcome approach through the community. And our study, or our survey, and our reports show that it is a model that works.

**Dr. McLemore:** Now that the federal dollars and the federal programs are going to sunset, do you believe this approach can be helpful in terms of both flu and COVID vaccine?

**Dr. Samoa:** 100%. So, we were able to demonstrate that there was high vaccine hesitancy in a lot of the subgroups. And a lot of the hesitancy was regional. We are able to demonstrate now, though, even with that hesitancy. Now, mind you, this is a group that should be very afraid of COVID because they have the highest rates of death and contraction in the country.

And yet you have a fair amount of hesitancy, even with that in the background. I think what was able to happen with our community leadership was they were able to overcome a lot of that hesitancy, and in a lot of those regions, the vaccination rates were over 90%. That is what I mean by starting with the community, because not only can they look at the assessment, but they can also overcome the obstacles that are brought about by that assessment. So, I hope that we learned something from COVID. That the other chronic conditions that have been pre-existing health disparities for NHPI communities, such as cancer and diabetes—you can apply this same approach to. Just because the urgency is not there, it does not mean that the rates are not high.

**Dr. Quach:** For us, why we wrote the article—when the pandemic hit, our communities experienced a dual pandemic of both COVID and racism. And our patients literally went underground. Asian Health Services is in Oakland, California, right in the heart of Chinatown, Little Saigon really amazing places, and all of a sudden it was empty. It was not just the fear of COVID, it was the fear of the violence. And for us as a community health center—as a federally qualified health center that is 48 years old, serving 50,000 patients in 14 different languages, these are on-site staff that are from the communities that we serve—we really saw that we had to transform our care literally within days to telehealth.

Because when we called up our patients, they were scared, they were committing suicide, they were depressed, and they could not see their doctors. We had to get them into telehealth, and it was so much work to do that. I do not think those stories are ever told. And part of the problem why we have this misleading narrative around the model minority myth, or that COVID only impacts black and brown communities, is because in part we are not sharing our stories.

So, we thought it was a call to action for us to write this up, and we did. Some of the key findings are its focus not just on the data disaggregation, but the compounded effects of language barriers in a time when everything went digital and our patients were left out. Like many other communities, they were left out on the sidelines, while they were blamed for the virus and ignored when it came to much needed services. We needed to tell that story. Some of our findings really showed that even in the payment system for health care providers, like ours, which is a community health center, they wanted us to switch over and everyone to be on video. Well, not all things are created equal. And it was important for us to advocate that they held on to audio visits. Being able to call someone on the phone was a lifeline for many of our patients, particularly the elderly limited English-proficient ones.

Then the other key thing that we said is, our community is very capable of learning some of the digital literacy, but it takes a lot of investment. It takes the workforce, like you were talking about, Dr. McLemore. It takes that bicultural and bilingual workforce to work with them. I do not think it is enough for us to say we will bring broadband, we will give you smartphones. It is not, and we showed that in our remote patient monitoring—that it took staffing that we had to set aside to really handhold them through this. But once they got it, they were able to convert. And to that question about how do we transform to this new digital era without the language—we cannot. We really need to advocate that not only is it in language, but it is also in the pictures.

For low literacy communities, we have the staffing to support them. Because as we have seen from the pandemic, the disparities were already existing. It just came out more. But for our community, that invisibility became worse. And it takes many community-based organizations, many community-based researchers, to really document that.

**Dr. McLemore:** Having come from Oakland, California and knowing your incredible work for a very long time, one of the things I loved about your piece was that telehealth does not mean there is no personnel attached to the health care.

And last, but not least, Dr. Zhang.

**Dr. Zhang:** Thanks, Monica. I really appreciate the opportunity. Your leadership, which as Grace mentioned, is extremely important in this time of the two pandemics affecting Asian Americans and Pacific Islanders. What motivated Adam and I thinking about the issue really coming together is, as everybody mentioned, the lack of data and evidence within the Asian American and Native Hawaiian Pacific Islander communities.

Many times, we lumped everything together for AANHPI and we have a good number, but we do not understand the deep-down reasons and the causes to the disparities within the community. That is probably also one reason I really like Dr. Samoa's piece working with the community, identifying the burdens, especially difficult situations within the local areas, regional areas. We will find out the resources to address specific disparities and have the most culturally and linguistically appropriate interventions to address these issues.

I think these are the evidence we needed in order to really change the policy. My previous work with U.S. Preventive Task Force made me realize that only if you have the data and evidence, then you will have the recommendations, then Medicare and Medicaid will pay for it. Without data within all the AANHPI communities we care, nothing will happen. So, congratulations to everybody for the great work here. I think we need more special issues on these topics, so we have enough data to really have community engaged efforts and have precision public health interventions tailored to specific populations.

**Dr. McLemore:** Your piece is another that struck me because as we think about other data sources and federal data sources—we resource allocate based on the census. There are so many different places within the federal government where we need to see transformation around data. Your piece was just spot-on in terms of calling that out in ways that I think are super important.

I think we need to think about other domains and other topics where *Health Equity* could lift this work up. And I do not think this is just the one and done first special edition.

I think that just happened to be the first, but we probably will have several others. One of the questions that came up that is like a question we had earlier, which is in addition to the overt acts of hate, are microaggressions in both personal and professional situations tracked? And if yes, what are the data telling us, and where and how can agencies—both federal, local, and state—address this?

I am looking at you, Dr. Samoa, maybe as somebody who might have some inkling around this, but maybe you do not. Not to put you on the spot.

**Dr. Samoa:** I do not know of any collection of microaggression at any kind of local or federal level. I know that it is up to individual institutions to do their own tracking. What their reporting mechanism of that tracking varies. I think that is one of the things that prohibits intervention, is the detailed tracking of these conditions.

I think there should be some type of national database where these types of events are reported. The other issue we are having is that the reporting of racist events is still problematic in that—and Dr. Thu can probably talk to this better than I can—is that there are a lot of communities that are not fully vested in their relationship with law enforcement, and yet they are asked to report vulnerable things to them.

I think a lot more education led by the Department of Justice needs to start to happen so that communities can start to foster that trustworthiness, if you will, so that they begin reporting at that level.

**Dr. McLemore:** Totally agree. I think Thu can answer this question, too, because you hinted at this in your comment, which is, in addition to language access, how does one address the issue of when a community member and/or patient is not proficient or has low literacy in their own native language? They can speak and understand it, but not necessarily read or write it.

Is there any focus on use or development of pictographs?

**Dr. Quach:** We continue to provide education not just in language, but a lot of in the forms of pictures and videos. We have been putting out videos just to help people know how to take a COVID test and how to do all of these other things. But I think it still speaks to the commitment to a bilingual and bicultural workforce. We can never take that away.

We cannot replace that. What I am fearful of is automation of everything now that is digital. So, I think we need to still advocate for investment in the workforce and in the communities—putting them in the communities that we serve.

**Dr. McLemore:** Completely agree. That is a perfect way to end the session. I'm going to turn it over to you, Dr. Chen, to close us out.

***Dr. Chen:* I just want to echo Dr. McLemore. Thank you all for participating, and thank you to the panelists for your contributions to this special issue for the discussion Dr. McLemore, we should talk and think about future special issues.**

**Dr. McLemore:** I agree. We want to be planning to make sure that we can have those tied up, because this is the first of what I hope to be many collaborations to highlight these important issues and to move us to health equity.

